# 

**Published:** 1895-09

**Authors:** 


### CONTENTS:

### SELECTIONS.

Article I.—The Adulteration of Drugs. By Willis G. Tucker, M. D., Ph. D. - - - - - - - - 193

" II.—Glass Filling, Jacket Crowns, Amalgam, Gold Caps, Bridges, etc.—A Scrap of Their History. By Wm. H. Trueman. - - - - - - 205

" III.—Anæsthesia vs Asphyxia. By S. A. Aykroyd, D.D.S. L. D. S. - - - - - - - 211

" IV.—Higher Dental Ethics. J. H. Smyser, D. D. S. - 218

" V.—Amalgam and How to Manipulate It. A. C. Hewitt. 225

### EDITORIAL, ETC.

American Dental Colleges. - - - - - - 227

Quackery Defined. - - - - - - - - 230

### MONTHLY SUMMARY.

Treating Nausea Caused by Taking Impressions. - - 230

Filling Pulpless Teeth with Fistulous Opening. - - - 231

Gold and Porcelain Crowns. - - - - - - 232

Decayed Teeth. - - - - - - - - 232

New Local Anæsthetic. - - - - - - - 233

Medicine and Dentistry. - - - - - - - 233

Hygiene of the Eyes. - - - - - - - 235

Infected Root Canals. - - - - - - - 235

Disinfectants. - - - - - - - - 236

How to Arrest a Boil, Carbuncle, or Malignant Pustule. - - 236

To Prepare Cavities. - - - - - - - 237

The Treatment of Epithelioma. - - - - - - 238

For Hemorrhage. - - - - - - - - 240

Metallurgy. - - - - - - - - - 249

